# Determinants of 50 m Front Crawl Performance in Adolescent Non-Elite Female Swimmers: A Longitudinal Study

**DOI:** 10.3390/jfmk10030274

**Published:** 2025-07-17

**Authors:** Mariusz Kuberski, Agnieszka Musial, Michalina Błażkiewicz, Jacek Wąsik

**Affiliations:** 1 Institute of Physical Culture Sciences, Jan Dlugosz University in Czestochowa, 42-201 Czestochowa, Poland; m.kuberski@ujd.edu.pl (M.K.); j.wasik@ujd.edu.pl (J.W.); 2 School of Biological and Behavioral Sciences, Queen Mary University of London, London E1 4NS, UK; 3 Faculty of Rehabilitation, The Jozef Pilsudski University of Physical Education in Warsaw, 00-968 Warsaw, Poland

**Keywords:** swimming, breaststroke measurements, longitudinal studies

## Abstract

**Objectives**: The aim of this study was to indicate which variables are the most important determinants of swimming results in the 50 m front crawl among non-elite pre-pubertal female swimmers. **Methods**: The study group consisted of 14 female swimmers (at the time of the research commencement—biological age: 10.52 ± 0.37 years; body mass: 34.99 ± 2.77 kg; height: 146.00 ± 3.05 cm). The study was conducted over three years. The swimmers performed capacity training recommended by the British Swimming Federation. Every 6 months, in the participants the following parameters were measured: percentage of body fat; anthropometric measurements; aerobic and anaerobic capacity; and respiratory parameters: vital capacity—VC, forced expiratory volume—FEV1, and forced vital capacity—FVC. Additionally, a 50 m front crawl swim test was performed. **Results**: After adjusting for multicollinearity, the most influential determinants of swimming performance were anthropometric measures: shoulder width was the most influential predictor, with a regression coefficient of −0.66, followed by foot length (with a beta of −0.15) and chest depth (with a beta of 0.008). The remaining anthropometric and physical predictors did not contribute to the prediction of 50 m freestyle performance. **Conclusions**: These research results suggest to coaches and trainers that sports performance in sprint distances in pre-pubertal girls is not determined by aerobic and anaerobic capacity or body fat but is based on the somatic build of the swimmer.

## 1. Introduction

Competitive swimming is divided into four swimming styles (front crawl, backstroke, butterfly stroke, and breaststroke) and race distances ranging from 50 m to 1500 m, emphasizing the importance of maintaining high levels of general endurance [1]. Athletic performance in swimming depends on numerous factors, including genetic predispositions encompassing innate morphological traits and appropriately tailored training loads during the training process [2,3,4]. In prepubescent children’s swimming training, it is essential to consider morphological and physiological differences that change throughout ontogenetic development. These differences include bone growth, muscle mass, fat tissue, motor skills, and the functioning of the respiratory and circulatory systems. A crucial recommendation for young swimmers’ training is adjusting the training program to the athlete’s biological age [5].

Researchers disagree on which factors most significantly influence performance in the 50 m front crawl in prepubescent children. Some studies suggest that somatic structure plays the most critical role [6], while others emphasize the importance of proper swimming technique for achieving high results [7]. There is evidence that somatic, energetic, and kinematic details of swimming technique create an individual performance model for each swimmer [8]. Other studies suggest that aerobic fitness in muscle energy production is associated with greater potential for high performance, not only in middle- and long-distance races but also in short-distance events. Research has shown that VO2max levels in young swimmers are, alongside biomechanical (functional) variables, among the strongest predictors of high performance in the 400 m freestyle race [9,10]. Cardiopulmonary efficiency, oxygen consumption, and energy production in more biologically mature young athletes are higher than those in their less developed peers, with differences ranging from 0.2 to 1.0 L⋅min^−1^ [11].

Studies also indicate that anaerobic capabilities in young swimmers are less developed than those in adult athletes, highlighting the significant influence of biological maturation on anaerobic capacity [12]. Biological maturity strongly determines strength–speed capabilities in females aged 13 to 14 years old and boys aged 14 to 15 years old [13]. An adequate level of strength enables more intense work, increases the ability to generate propulsive force, and is a crucial resource necessary for competing in sprint and middle-distance events, even in adolescence [14]. Specialized assessments, such as tethered swimming tests, have demonstrated that anaerobic processes play a key role in 50 m front crawl swimming performance among young swimmers [15]. Research indicates that swimming speed is the best indicator of swimming technique efficiency both before and during puberty [9]. Additional analyses have confirmed that swimming speed is a crucial measure of young swimmers’ technical advancement, particularly in 100 m and 400 m front crawl events [16]. It has also been emphasized that stroke length is one of the primary factors distinguishing the best swimmers aged 11 to 13 years old [7].

The findings of the cited studies demonstrate varied perspectives among researchers regarding the impact of different variables on 50 m front crawl performance in prepubescent children. Most studies have focused primarily on elite swimmers, meaning that they receive specialized training aimed at improving performance and are above-average swimmers. Selection into elite groups is also based on pre-selection. There is also a lack of longitudinal studies in the available literature, particularly in children. This highlights a significant gap in the research. This study focuses on girls who voluntarily enrolled in the proposed swimming training, regardless of their current skill level and body composition. The authors of this study want to show which of the analyzed variables in the pre-pubertal period will have the greatest impact on sports performance in the sprint distance among girls who do not belong to the sports elite. This will provide a more reliable representation of the influence of training and natural biological development on performance than the effects of initial selection [17].

Therefore, the authors propose the following research questions: (1) Which of the studied variables are the strongest predictors of 50 m front crawl performance in the examined swimmers? (2) How does the pace of change of the analyzed variables develop over 3 years in girls performing swimming training? In relation to these research questions, two hypotheses are put forward: (1) The most influential determinant of the result in the sprint distance will be anaerobic capacity. (2) The authors assume that the results for the analyzed variables will change after 3 years of research. The answers to these questions will allow us to verify whether it is justified to conduct pre-selection for swimming training among pre-pubertal girls and to show how the development of individual features shapes performance in girls aged 10–12.

## 2. Materials and Methods

### 2.1. Characteristics of the Study Group

This study included a cohort of 14 adolescent female swimmers (mean biological age: 10.52 ± 0.37 years, mean body mass: 34.99 ± 2.77 kg, mean height: 146.00 ± 3.05 cm at baseline) who trained in student sports clubs in Czestochowa, Poland. The selection criteria were age at the beginning of the study (10 years), beginning of swimming training in a student sports club in the current year, a certificate of no contraindications to swimming (accepted by the bioethics committee), and the consent of legal guardians to participate in the experiment. Recruitment into the sports clubs occurred without preliminary selection. At the commencement of this study, the participants initiated structured swimming training, having already acquired basic swimming skills through biweekly swimming lessons. According to information provided by the legal guardians, apart from compulsory physical education lessons at school, the study group did not participate in any other sports training, and all participants from the experimental group remained in the premenstrual stage throughout the six study phases. According to the Participant Classification Framework proposed by McKay et al. [18], the swimming group in this study can be classified as Tier 2: Trained/Developmental.

Following the Declaration of Helsinki, all participants and their parents were informed of the study’s purpose and methodology. Written informed consent was obtained from both the participants and their legal guardians. The study protocol received approval from the Bioethics Committee for Scientific Research at Jan Dlugosz University in Czestochowa, Poland. (approval number KB-2/2012).

### 2.2. Longitudinal Study Design

**Figure 1:**
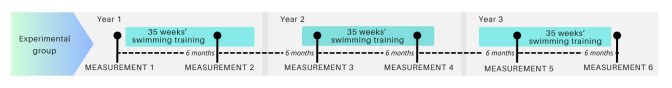
Study design diagram.

This research was an experimental longitudinal study. The study was conducted over three consecutive years, from autumn 2011 to spring 2014, with measurements taken every six months, between 8:00 AM and 12:00 PM (a total of six measurements) (Figure 1).

The swimmers’ training macrocycle was designed based on the British Swimming Federation guidelines for females aged 9–12 years [19]. It consisted of four training sessions per week, held in the morning (6:30–7:40 AM). Each training session lasted 70 min, during which swimmers performed several sets of sections (Table 1). The average daily swimming distance was approximately 1500 m in the first year, 2000 m in the second year, and 2500 m in the third year. Each session began with a 10 min warm-up on land, followed by a warm-up in the water, swimming 200 to 400 m in front crawl or backstroke.

Emphasis was placed on body position, the effectiveness of arm strokes and leg work, and maintaining the correct “swimming step”. Turns and swimming underwater were also refined. To improve technical elements, coaches used specialized swimming equipment, including short and long fins, swimming paddles, and resistance bands. Aerobic capacity was primarily developed by swimming front crawl. The training sessions concluded with stretching exercises on land, lasting about 7 min. These stretching exercises enhanced the range of motion in the shoulder girdle and increased ankle joint mobility.

### 2.3. Biological Age and Anthropometric Measurements

Body mass and height were assessed using a scale with a stadiometer (WPT 150.0; RadWag, Radom, Poland) with a precision of 0.1 kg and 0.5 cm, respectively. Biological age was determined using the following formula [20], where body mass age and body height age were estimated using Pirquet’s tables for females from the Lubusz region [21], and chronological age was calculated based on Jopkiewicz 1998 [22].

Biological age = (body mass age + body height age + chronological age)/3

body mass/height age—estimated using Pirquet’s tables
chronological age—time between date of birth and the date of measurement

The following anthropometric measurements were taken: body mass, height (B–v), chest depth (xi–ths), chest width (thl–thl), shoulder width (a–a), hip width (ic–ic), upper limb length (a–da), hand width (mu–mr), lower limb length (B–sy), foot width (mtt–mtf), and foot length (pte–ap) [23]. Based on the body mass and height, the BMI (body mass index) was calculated: BMI = body mass (kg)/height^2^. Anthropometric measurements were taken on the right side of the subject’s body in a standing position (Frankfurt plane) using a spreading caliper with an accuracy of 1 mm. All measurements were taken between 8:00 a.m. and 12:00 p.m.

### 2.4. Body Fat Measurements

The body fat percentage was assessed by measuring the skinfold thickness at four anatomical sites: over the biceps, over the triceps, beneath the inferior angle of the scapula (referred to as the shoulder blade), and above the iliac crest (referred to as the stomach). All measurements were taken on the right side of the body in a standing position (Frankfurt plane) using a Harpenden skinfold caliper (M2 TOP, Käfer, Bremen, Germany) with a precision of 0.1 mm. Based on the skinfold measurements, the body fat percentage (% Body fat) was calculated using the formula provided by Slaughter et al. [24]. If the sum of the triceps and subscapular skinfolds was ≤35 mm, the equation used was

% Body fat = [1.33 × (R + L)] − [0.013 × (R + L)^2^] − 2.5

R—skinfold over the triceps
L—skinfold beneath the scapula

If the sum of the triceps and subscapular skinfolds was >35 mm, the equation used was

% Body fat = 0.546 × (R + L) + 9.7

R—skinfold over the triceps
L—skinfold beneath the scapula

### 2.5. Aerobic and Anaerobic Capacity Measurements

The Maximal Multistage 20 m Shuttle Run Test (commonly referred to as the Beep Test) was used to measure aerobic capacity [25]. This test involved running back and forth over a 20 m distance, with the pace controlled by audio signals. The participants had to complete the distance within the time allotted by the sound, which gradually shortened with each subsequent stage. The initial running speed was 8.5 km/h, and it increased by 0.5 km/h at each stage. The number of shuttle runs also increased with each stage: the first stage required seven runs at a consistent pace, the second stage required eight runs, and so on, up to the fourth stage. From stages five to eight, participants completed 10 runs, and from stages nine to thirteen, 12 runs of 20 m were required. If a participant failed to reach the line before the next sound, their attempt was terminated. The total number of successful shuttle runs was recorded. Based on the speed at which the last stage was completed and the participant’s calendar age, maximal oxygen uptake (VO2max), an indicator of aerobic fitness, was calculated using the formula provided by Léger et al. [26]:

$$\overset{\cdot }{V}O2\;\max=31.025+3.238\times P-3.248\times W+0.1536\times P\times W$$

P—maximum running speed (km/h) from the last completed stage
W—calendar age, rounded down to the nearest whole number

The Standing Reach Jump test was performed with the subject standing sideways (right or left, depending on whether they were right-handed or left-handed) against a wall, with the arm extended as high as possible. The height reached by the subject’s raised right or left upper limb was marked. Then, the subject, by bending their lower limbs at the knee joints to a 90-degree angle, performed a vertical jump upwards, using an arm swing, and marked the height they were able to reach with their upper limb. The test was conducted three times, without shoes, and the best result was used for analysis. Based on the difference in height, the subject’s body mass, and the gravitational acceleration value, the maximum anaerobic work (MPA) was calculated, which served as an indicator of anaerobic capacity [27].

MPA = m × g × h

m—body mass in kilograms
g—represents gravitational acceleration of 9.81 m/s^2^
h—represents the jump height in meters

### 2.6. Respiratory Measurements

The measurement of respiratory volumes was performed using a VF-S spirometer (PELAB, Poland) and included determination of the VC (vital capacity), FVC (forced vital capacity), and FEV1 (forced expiratory volume).

To obtain the VC measurement, after several minutes of calm breathing in a seated position, the subject assumed a standing position and then took the deepest possible inhalation, followed by expelling the maximum amount of air into the spirometer (for at least 6 s). The test was conducted with a nose clip applied to the subject. The procedure was repeated three times (with a 5 min break between trials), and the best result was taken into account.

The procedure for measuring FEV1 was similar, but after taking a standing position, the subject performed a forced exhalation, during which they were instructed to expel as much air as possible from the lungs within one second.

In order to measure FVC (forced vital capacity)—the total volume of air forcefully exhaled after a maximal inhalation after taking a standing position—the subject took a deep breath in and then forcefully exhaled into the mouthpiece as quickly and completely as possible.

### 2.7. Statistical Analysis

All statistical analyses were conducted using R (version 4.0.4) [28]. Descriptive statistics, including means and standard deviations, were computed for all anthropometric and performance variables across the six measurement time points. To assess changes over time, a repeated-measures analysis of variance (ANOVA) was performed to determine whether significant differences existed between the first and final measurements for each variable. Skew statistics were examined to assess normality, with a threshold of −1/1. To investigate the relationship between anthropometric and physiological variables and 50 m crawl performance, Pearson correlation coefficients were computed between each predictor and the swim score.

Multivariable prediction of the 50 m crawl score was performed using an elastic net regression model, which incorporates both L1 (lasso) and L2 (ridge) regularization to address the issues of multicollinearity and overfitting [29]. We performed the elastic net analyses using the R package glmnet (version 4.5.0) [30], implemented in caret [31]. The elastic net model was selected due to the high degree of correlation among anthropometric variables, which could lead to redundancy in predictor contributions. Elastic net regularization tries to minimize the following loss function [32]:

||y − Xβ||2 + λ(α × |β|1 + (1 − α) × |β|2)

||y − Xβ||2—residual sum of squares
|β|2—sum of the squared betas (the L2 penalty)
|β|1—sum of the absolute betas (the L1 penalty)
X—N × P (‘N’ observations and ‘P’ predictors) matrix of anthropometric and performance predictors

The elastic net model was trained using 10-fold cross-validation to optimize the tuning parameters and avoid overfitting. Variables with regression weights of zero were considered non-contributory to the model. The proportion of variance explained by the model (R^2^) was used to evaluate predictive accuracy, with a focus on identifying the most influential predictors of 50 m crawl performance. Standardized regression coefficients are reported to facilitate the interpretation of the relative importance of each predictor.

Specifically, prior to study initiation, we estimated the required sample size using G*Power 3.1.9.2, assuming a significance level of α = 0.05, power = 0.95, and effect size f = 0.6 (based on the existing literature and expected differences). This analysis indicated that a minimum of 12 participants per group would be sufficient.

## 3. Results

### 3.1. Descriptive Statistics

Descriptive statistics for each variable computed across the six measurement time points are presented in Table 2.

**Figure 2:**
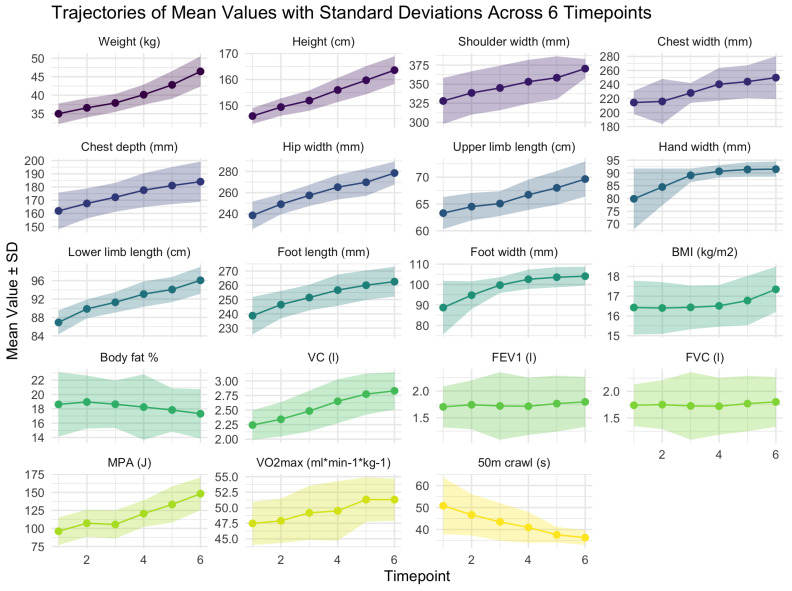
Trajectories of anthropometric and performance measurements across the 6 follow-up timepoints. Note: SD—standard deviation; BMI—body mass index; VC—vital capacity; FEV1—forced expiratory volume in 1 s; FVC—forced vital capacity; MPA—maximum peak acceleration. All anthropometric variables were measured in mm, apart from upper and lower limb length, which were measured in cm.

Based on the skew statistics, none of the variables displayed a severe skew. The mean trajectories of the anthropometric and performance measures are presented in Figure 2.

ANOVA tests were conducted to determine whether there were statistically significant differences between the first and final measurements. The results of the ANOVA are presented in Table 3. Significant differences were observed in all anthropometric and performance measures, with the exception of BMI, body fat percentage, FEV1, and forced vital capacity (FVC).

### 3.2. Multivariable Prediction of 50 m Crawl Score

**Figure 3:**
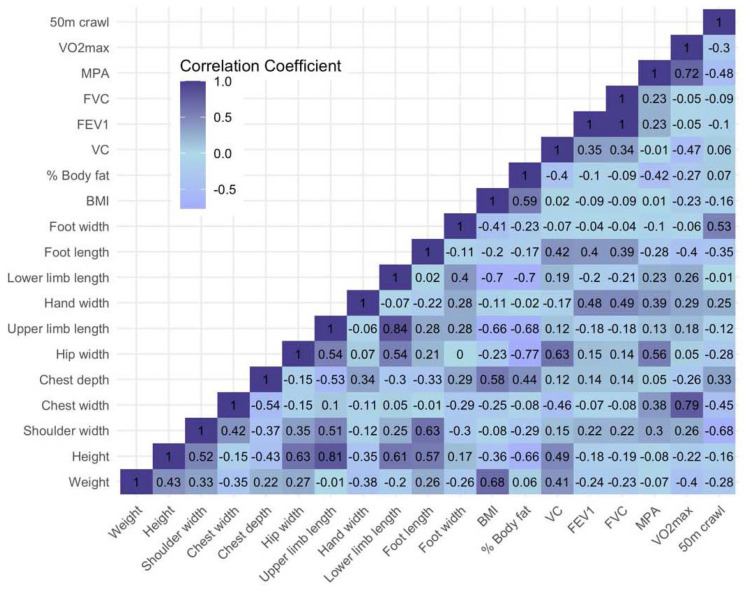
Heatmap of correlations between anthropometric and performance measures and 50 m crawl score.

Correlations between predictors and the 50 m crawl score are presented in Figure 3. The average correlation for the anthropometric variables was observed to be moderate and negative, with values ranging from r = −0.28 for weight to r = −0.68 for shoulder width. The strongest negative correlation was observed for shoulder width (r = −0.68), followed by chest width (r = −0.45). In contrast, foot width showed the strongest positive correlation with swimming performance (r = 0.53). The measures BMI and body fat percentage showed weak correlations with the 50 m crawl score, with values ranging from r = −0.07 to r = 0.07. Lung function variables like VC, FEV1, and FVC also presented weak to moderate correlations, with the strongest being r = 0.41 observed for VC.

The elastic net regression model accounted for 44.3% of the total variance in the 50 m crawl performance. After accounting for multicollinearity, shoulder width was the most influential predictor, with a regression coefficient of −0.66. The other two significant predictors were foot length (with a beta of −0.15) and chest depth (with a beta of 0.008). The remaining anthropometric and physical predictors did not contribute to prediction of the 50 m crawl score. The model fit appears suboptimal, as indicated by the relatively high RMSE of 2.82, suggesting substantial prediction error.

## 4. Discussion

The research hypothesis of this study assumed anaerobic capacity as the most influential factor influencing sprint performance. This hypothesis was not confirmed, and the results of this study indicate anthropometric parameters as the most important determinants of the result for front crawl at a distance of 50 m.

The most influential predictor of performance in the 50 m front crawl was shoulder width. The authors of other research studies agree that swimming training during the prepubescent period leads to an increase in shoulder width and decrease in hip width, contributing to the development of a body shape that is thought to be “typical for swimmers.” In front crawl, high swimming speed primarily depends on the ability to generate maximum propulsion force, which is necessary to overcome resistance, especially with the arms. The propulsion force, which drives the swimmer’s body forward, is created by the arms when they push the water backward [33]. For elite adult swimmers, studies have shown a correlation between the individual peak isokinetic force moment of the arms and 50 m freestyle swimming performance. Other authors have noted that the swimming speed in front crawl for sprint distances depends on the speed with which the arm cycle is repeated [34]. In our experiment, the swimmers were subjected to endurance training, in which they frequently performed arm work specifically for the front crawl. This may have led to an increase in shoulder width, which had the most significant impact on the 50 m crawl performance. The impact of shoulder width on sports performance should mainly be associated with the 50 m distance, where, in a 25 m pool, there would be only one turn, followed by an underwater phase. However, for longer distances, wide shoulders may increase the drag experienced underwater during the butterfly kick phase, particularly in the “torpedo position,” as discussed by researchers focusing on this area [35].

The second most influential predictor of performance in the 50 m breaststroke was foot length. Intense leg work contributes to achieving the highest speeds necessary to meet training goals or compete in sprint distances [33]. It is important to note that the key factor is not just the strength and speed of leg movements but the effectiveness of the kick. This depends primarily on foot and ankle flexibility, as well as foot length [36]. Foot length is also important for maintaining the correct position at the water’s surface, which prevents the swimmer from assuming an increasingly vertical body position that generates significant drag while moving through the water [37]. The importance of leg work in swimmers increases as the distance shortens. In sprint races, swimmers perform six leg kicks for each arm cycle. This makes legwork a crucial element in short-distance events. In a study by Morouco et al. [38], the authors found that young female swimmers engaged their legs more to increase speed in the 50 m front crawl compared to males, who achieved greater speeds by using faster arm movements. It is also worth noting the impact of foot length on swimming underwater with butterfly kicks. As we know, after the start and turn in the 50 m race, swimmers perform powerful two-legged kicks in the butterfly style. A longer foot, with proper technique, generates greater speed, allowing the swimmer to move more quickly underwater [39]. Based on our research, it can be cautiously concluded that foot length should be considered by coaches and instructors when selecting swimmers for the sport.

The third variable influencing performance in the 50 m front crawl was chest depth. The authors attribute the presence of this variable to breathing exercises performed by young swimmers during the prepubescent period. It is known that better lung parameters are influenced by the environment in which swimming training primarily takes place. Difficult exhalation into the water, as well as the horizontal body position, increases the flexibility and mobility of the chest [40]. The authors emphasize that during swimming training, young female swimmers show improvements in respiratory parameters, which, in turn, enhance their performance in both long and short distances. This is due to the long physical efforts performed with the chest in a horizontal position, influenced by varying hydrostatic pressure, which is associated with periods of breath-holding that lead to regular, temporary hypoxia [41].

The elastic net model showed that many physical characteristics, such as weight, height, BMI, and VO2max, had weights of zero, suggesting that these variables did not significantly contribute to explaining the variation in 50 m crawl performance. This outcome likely reflects the presence of multicollinearity, where these predictors are highly correlated with other variables in the model, leading to redundant contributions. For example, variables such as height and weight, which are often correlated with each other and with other body measurements, may have been effectively “captured” by other, more influential predictors, such as shoulder width and foot length. The elastic net method, which combines both ridge and lasso regularization, helps to address multicollinearity by shrinking the coefficients of redundant predictors toward zero, resulting in a more parsimonious model.

Our research indicates that all analyzed anthropometric variables improved over the course, which was consistent with our expectations. Additionally, variables such as VO2max, MPA, and FVC increased over the three-year swimming training period. However, no significant changes were observed in BMI, the percentage of body fat, FEV1, or VC. Therefore, the hypothesis regarding changes in the studied variables during the 3-year study was partially confirmed.

The lack of changes in BMI and the percentage of body fat should be interpreted with caution. While BMI allows for the assessment of potential body mass abnormalities, it does not provide information about the ratio of fat mass to lean body mass. In elite adult swimmers, beneficial changes in body composition can be observed after just one month of aerobic training [42]. Additional physical activity, such as swimming, plays a crucial role in preventing excessive weight gain [43], which is particularly desirable in swimming sports.

A lower body weight may contribute to reduced resistance during the underwater phase, which impacts swimming efficiency [44]. Other studies have shown that young swimmers, compared to a control group, exhibited a lower percentage of body fat over a three-year training period. However, these athletes were selected for the swimming sport, which favors a lean body composition [40]. On the other hand, some studies indicated that young swimmers had a slightly higher percentage of body fat compared to a non-swimming group [45].

At the age of 13, fat tissue increased in the swimming group, which the authors attribute to the natural, physiological increase in body fat during puberty [46]. Based on our findings, although there were no statistically significant changes in BMI or the percentage of body fat among the swimmers over the three years, it can be cautiously concluded that swimming may be an effective way to prevent an increase in body fat, thus serving as an effective method in the fight against obesity. It is worth noting that one limitation of our experiment was the lack of control over the participants’ diet and other forms of physical activity during the study, which could have influenced the results.

Most research studies related to pulmonary parameters have demonstrated that both in swimming and in other endurance sports, over a three-year training period, VC and FEV1 improve significantly [47,48]. In swimming, VC is often higher from the outset of training due to the selection of taller individuals for the sport. Additionally, it has been shown that one year of intense endurance swimming training leads to significant improvements in both static and dynamic lung volumes, particularly enhancing flow volume in pre-pubescent swimmers [40]. These studies suggest that respiratory muscles during intensive endurance swimming training can increase in strength and endurance in response to specific training conditions in an aquatic environment. Such adaptations may be the result of specific environmental constraints affecting the pulmonary system. The basis of this interaction is often described by five water stressors: breathing under pressure, prolonged exhalation underwater, restricted breathing patterns, altered gas tensions in the alveoli, repeated lung expansion to total lung volume, the prone position, and intensive early childhood training programs [49]. As a result, minute ventilation is reduced, and swimmers’ lungs must accommodate large gradients in partial pressures of CO_2_ and O_2_. This leads to an increase in the lungs’ diffusing capacity. The high adaptive capacity of healthy lungs and the stronger response of younger individuals to stimuli promoting growth may be strong indicators of the positive effects of early swimming training, explaining the greater respiratory capacity in swimmers. Holding one’s breath for extended periods, especially during underwater swimming (e.g., after a start jump and turns), contributes to the development of respiratory muscles, including the diaphragm. The higher pressure resulting from immersion in water improves the chest wall’s elasticity, leading to higher lung function [50]. In studies where no significant changes were observed over three years, the authors attributed this to the lack of selection for swimming, noting that, as we know from the literature, taller individuals are preferred in swimming [45]. Additionally, training focused solely on endurance tasks over a three-year period might lead to smaller changes in respiratory parameters compared to training involving tasks from different energy systems [51].

## 5. Conclusions

The results of this study suggest that swimming coaches and instructors should approach the initial selection with great caution because, in pre-pubescent girls who are not elite swimmers, performance does not depend on physiological parameters such as aerobic and anaerobic capacity, respiratory parameters, or body fat. The most important factor influencing their performance is the somatic structure of the body. Further research in this area, including a larger population and taking into account the diets of swimmers, may provide more information on other characteristics that may affect 50 m freestyle sprint performance in girls. Conducting similar studies in an older group (post-pubertal age) will provide information on how determinants of swimming performance change over time. It is worth considering further studies that include monitoring freestyle technique using appropriate laboratory equipment.

## 6. Study Limitations

The study has several limitations that should be taken into account when interpreting the results. The study group was relatively small, which limits the generalizability of the results to a wider population. A comparison with a control group would certainly have facilitated the assessment of the impact of training and natural biological changes. Factors such as the participants’ diets, motivations, and environmental conditions were not taken into account. Aerobic capacity was assessed via a running test rather than a swimming test due to parental consent restrictions. Also, tests performed outside an aquatic environment may constitute a certain limitation of the research project performed. The variety of parameters measured was limited, which precludes an analysis of more detailed technical and motor aspects. In addition, the participants came from local sports clubs in Poland, which may limit the representativeness of the results.

## Figures and Tables

**Table 1:** Swimmers’ training macrocycle as per British Swimming Federation guidelines for females aged 9–12.

Research Time	Number of Trainings	Training Unit Diagram	Training Unit Diagram
Year 1 (35 weeks)	4 training sessions per week	Warm up: 200–300 m Main part: 5 × 50 m only arms 5 × 50 m only legs 5 × 50 m coordination arms/legs 2 × 100 m full style Cool down: 200–300 m	1500 m in 1 training session
Year 2 (35 weeks)	4 training sessions per week	Warm up: 300–400 m 6 × 50 m only arms 6 × 50 m only legs 6 × 50 m coordination arms/legs 5 × 100 m full style Cool down: 200–300 m	2000 m in 1 training session
Year 3 (35 weeks)	4 training sessions per week	Warm up: 300–400 m 5 × 100 m only arms 5 × 100 m only legs 5 × 100 m coordination arms/legs 4 × 100 m full style Cool down: 200–300 m	2500 m in 1 training session

**Table 2:** Descriptive statistics and results of the ANOVA between the first and final measurements.

Measurement 1									Measurement 6								ANOVA		
Variable	Mean	SD	Median	Min	Max	Range	Skew	Kurtosis	Mean	SD	Median	Min	Max	Range	Skew	Kurtosis	F-Value	df	*p*-Value
Weight [kg]	35.00	2.77	34.80	31.40	40.10	8.70	0.33	−1.39	46.40	4.09	44.60	42.40	55.60	13.20	1.04	−0.41	−1.35	75.20	<0.001
Height [cm]	146.00	3.04	145.00	141.00	152.00	11.00	0.33	−0.94	164.00	5.31	164.00	156.00	176.00	20.50	0.72	0.15	0.32	116.00	<0.001
Shoulder width [mm]	328.00	30.10	333.00	244.00	358.00	114.00	−1.36	1.56	370.00	12.60	373.00	351.00	389.00	38.00	−0.04	−1.50	−1.21	23.50	<0.001
Chest width [mm]	214.00	16.60	212.00	182.00	240.00	58.00	−0.09	−0.87	250.00	30.40	242.00	217.00	339.00	122.00	1.70	2.53	0.26	14.70	<0.001
Chest depth [mm]	162.00	13.80	164.00	136.00	183.00	47.00	−0.64	−0.63	184.00	15.20	184.00	159.00	213.00	54.00	0.26	−0.99	−0.48	16.10	<0.001
Hip width [mm]	238.00	13.10	242.00	210.00	255.00	45.00	−0.80	−0.58	278.00	10.90	276.00	265.00	305.00	40.00	0.88	0.09	−1.47	77.50	<0.001
Upper limb length [cm]	63.30	3.00	63.50	59.00	68.00	9.00	0.03	−1.43	69.60	3.27	69.50	65.00	75.00	10.00	0.09	−1.38	−1.09	28.70	<0.001
Hand width [mm]	79.90	11.90	86.00	55.00	91.00	36.00	−0.85	−0.85	91.50	2.98	91.00	87.00	95.00	8.00	−0.05	−1.70	−1.60	12.60	0.001
Lower limb length [cm]	86.90	2.62	87.50	81.00	90.00	9.00	−0.77	−0.59	96.10	2.95	95.50	92.00	102.00	10.00	0.35	−1.03	−0.75	75.40	<0.001
Foot length [mm]	239.00	13.10	238.00	202.00	257.00	55.00	−1.22	1.84	263.00	10.40	262.00	246.00	282.00	36.00	0.26	−1.14	−1.80	28.30	<0.001
Foot width [mm]	88.70	13.10	94.50	60.00	102.00	42.00	−0.94	−0.57	104.00	4.63	104.00	98.00	112.00	14.00	0.29	−1.40	−1.01	17.10	<0.001
BMI [ kg/m^2^]	16.40	1.36	16.20	14.30	18.30	4.03	0.17	−1.47	17.30	1.14	17.30	15.80	19.70	3.94	0.61	−0.58	−0.92	3.75	0.064
Body fat [%]	18.60	4.50	17.70	10.80	26.70	16.00	0.17	−1.10	17.30	3.45	16.70	11.40	22.60	11.20	0.05	−1.43	−0.75	0.74	0.396
VC [l]	2.24	0.25	2.23	1.80	2.78	0.98	0.23	−0.42	2.83	0.32	2.90	2.33	3.44	1.11	0.33	−0.98	−1.21	28.80	<0.001
FEV1 [l]	1.70	0.38	1.76	1.09	2.45	1.36	0.02	−0.93	1.80	0.47	1.76	0.98	2.52	1.54	0.08	−1.21	−1.36	0.34	0.568
FVC [l]	1.74	0.39	1.84	1.09	2.48	1.39	−0.07	−1.04	1.80	0.47	1.76	0.99	2.53	1.54	0.09	−1.22	−1.37	0.15	0.7
MPA [J]	96.10	19.10	99.80	62.90	126.00	63.00	−0.49	−1.02	148.00	22.70	146.00	118.00	193.00	74.40	0.37	−1.11	−0.94	596.00	<0.001
VO2max [mL·min^−1^·kg^−1^]	47.50	3.47	46.30	41.50	53.40	11.90	0.28	−0.88	51.30	3.39	52.30	43.80	55.60	11.80	−0.77	−0.46	−0.29	8.69	0.007
50 m crawl [s]	50.80	12.90	46.10	37.80	79.00	41.20	0.93	−0.52	36.30	3.37	35.60	31.90	45.10	13.30	1.13	0.81	0.35	16.50	<0.001

SD—standard deviation; BMI—body mass index; VC—vital capacity; FEV1—forced expiratory volume in 1 s; FVC—forced vital capacity; MPA—maximum peak acceleration; VO2max—maximal oxygen uptake.

**Table 3:** Elastic net model fit indices.

Metric	Value
N	14
R-squared	0.44
RMSE (95% CIs)	2.82 (1.76; 4.95)
MAE	2.13
AIC	149.20
BIC	161.34

N—sample size; RMSE—root-mean-square error; MAE—Mean Absolute Error; AIC—Akaike Information Criterion; BIC—Bayesian Information Criterion.

## Data Availability

The data presented in this study are available on request from the corresponding author. The data are not publicly available due to ongoing data collection and further research being conducted on this topic.

## References

[1] Eider P. (2015). Research Analysis of Selection Criteria at the Initial Stage of Swimming Training of Primary School Junior Students. Cent. Eur. J. Sport. Sci. Med..

[2] Ruiz-Navarro J.J., Cuenca-Fernández F., Sanders R., Arellano R. (2022). The Determinant Factors of Undulatory Underwater Swimming Performance: A Systematic Review. J. Sports Sci..

[3] Kuberski M., Polak A., Szołtys B., Markowski K., Zarzeczny R. (2022). Associations between Selected Biological Features and Absolute and Relative Swimming Performance of Prepubescent Boys over a 3-Year Swimming Training Program: A Longitudinal Study. J. Hum. Kinet..

[4] Kuberski M., Góra T., Wąsik J. (2024). Changes in Selected Somatic Indices in 10–12 Year Old Girls under the Influence of 3-Year Swimming Training. Phys. Act. Rev..

[5] Charmas M., Gromisz W. (2019). Effect of 12-Week Swimming Training on Body Composition in Young Women. Int. J. Env. Res. Public Health.

[6] Figueiredo P., Silva A., Sampaio A., Vilas-Boas J.P., Fernandes R.J. (2016). Front Crawl Sprint Performance: A Cluster Analysis of Biomechanics, Energetics, Coordinative, and Anthropometric Determinants in Young Swimmers. Mot. Control.

[7] Barbosa T.M., Bartolomeu R., Morais J.E., Costa M.J. (2019). Skillful Swimming in Age-Groups Is Determined by Anthropometrics, Biomechanics and Energetics. Front. Physiol..

[8] Lätt E., Jürimäe J., Mäestu J., Purge P., Rämson R., Haljaste K., Keskinen K.L., Rodriguez F.A., Jürimäe T. (2010). Physiological, Biomechanical and Anthropometrical Predictors of Sprint Swimming Performance in Adolescent Swimmers. J. Sports Sci. Med..

[9] Jürimäe J., Haljaste K., Cicchella A., Lätt E., Purge P., Leppik A., Jürimäe T. (2007). Analysis of Swimming Performance from Physical, Physiological, and Biomechanical Parameters in Young Swimmers. Pediatr. Exerc. Sci..

[10] Zacca R., Wenzel B.M., Piccin J.S., Marcilio N.R., Lopes A.L., de Souza Castro F.A. (2010). Critical Velocity, Anaerobic Distance Capacity, Maximal Instantaneous Velocity and Aerobic Inertia in Sprint and Endurance Young Swimmers. Eur. J. Appl. Physiol..

[11] Lätt E., Jürimäe J., Haljaste K., Cicchella A., Purge P., Jürimäe T. (2009). Physical Development and Swimming Performance during Biological Maturation in Young Female Swimmers. Coll. Antropol..

[12] Bencke J., Damsgaard R., Saekmose A., Jørgensen P., Jørgensen K., Klausen K. (2002). Anaerobic Power and Muscle Strength Characteristics of 11 Years Old Elite and Non-elite Boys and Girls from Gymnastics, Team Handball, Tennis and Swimming. Scand. J. Med. Sci. Sports.

[13] Boguszewski D., Stępień M., Adamczyk J.G. (2023). The Influence of Core Stability Exercises Programme on the Functional Limitations of the Musculoskeletal System in Girls Practising Volleyball. Phys. Act. Rev..

[14] Kalva-Filho C., Zagatto A., da Silva A., Castanho M., Gobbi R., Gobatto C., Papoti M. (2017). Relationships among the Tethered 3-Min All-Out Test, MAOD and Swimming Performance. Int. J. Sports Med..

[15] Geladas N.D., Nassis G.P., Pavlicevic S. (2005). Somatic and Physical Traits Affecting Sprint Swimming Performance in Young Swimmers. Int. J. Sports Med..

[16] Mezzaroba P.V., Machado F.A. (2014). Effect of Age, Anthropometry, and Distance in Stroke Parameters of Young Swimmers. Int. J. Sports Physiol. Perform..

[17] Bielec G., Peczak-Graczyk A., Waade B. (2013). Do Swimming Exercises Induce Anthropometric Changes in Adolescents?. Issues Compr. Pediatr. Nurs..

[18] McKay A.K.A., Stellingwerff T., Smith E.S., Martin D.T., Mujika I., Goosey-Tolfrey V.L., Sheppard J., Burke L.M. (2022). Defining Training and Performance Caliber: A Participant Classification Framework. Int. J. Sports Physiol. Perform..

[19] Lang M., Light R. (2010). Interpreting and Implementing the Long Term Athlete Development Model: English Swimming Coaches’ Views on the (Swimming) LTAD in Practice. Int. J. Sports Sci. Coach..

[20] Przewęda R., Ulatowski T. (1971). Ocena Wieku Rozwojowego. Teoria i Metodyka Sportu.

[21] Malinowski A., Asienkiewicz R., Tatarczuk J., Stuła A., Wandycz A. (2005). Dziecko Lubuskie.

[22] Jopkiewicz A., Suliga E. (1998). Biologiczne Podstawy Rozwoju Człowieka.

[23] Drozdowski Z. (2022). Antropologia Dla Nauczycieli Wychowania Fizycznego.

[24] Slaughter M.H., Lohman T.G., Boileau R.A., Horswill C.A., Stillman R.J., Van Loan M.D., Bemben D.A. (1988). Skinfold Equations for Estimation of Body Fatness in Children and Youth. Hum. Biol..

[25] Kasai D., Tsiros M.D., Eston R., Parfitt G. (2023). Ratings of Perceived Exertion from a Submaximal 20-m Shuttle Run Test Predict Peak Oxygen Uptake in Children and the Test Feels Better. Eur. J. Appl. Physiol..

[26] Léger L.A., Mercier D., Gadoury C., Lambert J. (1988). The Multistage 20 Metre Shuttle Run Test for Aerobic Fitness. J. Sports Sci..

[27] Van Praagh E. (2007). Anaerobic Fitness Tests: What Are We Measuring?. Med. Sport Sci..

[28] (2021). R Core Team: A Language and Environment for Statistical Computing.

[29] Zou H., Hastie T. (2005). Regularization and Variable Selection Via the Elastic Net. J. R. Stat. Soc. Ser. B Stat. Methodol..

[30] Hastie T., Qian J., Tay K. (2023). An Introduction to Glmnet. https://cran.r-project.org/web/packages/glmnet/vignettes/glmnet.pdf.

[31] Kuhn M. (2008). Building Predictive Models in R Using the Caret Package. J. Stat. Softw..

[32] Allegrini A.G., Karhunen V., Coleman J.R.I., Selzam S., Rimfeld K., von Stumm S., Pingault J.-B., Plomin R. (2020). Multivariable G-E Interplay in the Prediction of Educational Achievement. PLoS Genet..

[33] Deschodt V.J., Arsac L.M., Rouard A.H. (1999). Relative Contribution of Arms and Legs in Humans to Propulsion in 25-m Sprint Front-Crawl Swimming. Eur. J. Appl. Physiol. Occup. Physiol..

[34] Wakayoshi K., Yoshida T., Ikuta Y., Mutoh Y., Miyashita M. (1993). Adaptations to Six Months of Aerobic Swim Training. Int. J. Sports Med..

[35] Liu C., Xu B., Wan K., Sun Q., Wang R., Feng Y., Shao H., Liu T., Wang R. (2024). Improved Prediction of Swimming Talent through Random Forest Analysis of Anthropometric and Physiological Phenotypes. Phenomics.

[36] Miller D.I. (1975). Biomechanics of Swimming. Exerc. Sport. Sci. Rev..

[37] Papic C., McCabe C., Gonjo T., Sanders R. (2023). Effect of Torso Morphology on Maximum Hydrodynamic Resistance in Front Crawl Swimming. Sports Biomech..

[38] Morouço P.G., Marinho D.A., Izquierdo M., Neiva H., Marques M.C. (2015). Relative Contribution of Arms and Legs in 30 s Fully Tethered Front Crawl Swimming. Biomed. Res. Int..

[39] Veiga S., Lorenzo J., Trinidad A., Pla R., Fallas-Campos A., de la Rubia A. (2022). Kinematic Analysis of the Underwater Undulatory Swimming Cycle: A Systematic and Synthetic Review. Int. J. Env. Res. Public Health.

[40] Knechtle B. (2014). Relationship of Anthropometric and Training Characteristics with Race Performance in Endurance and Ultra-Endurance Athletes. Asian J. Sports Med..

[41] Silvestri M., Crimi E., Oliva S., Senarega D., Tosca M.A., Rossi G.A., Brusasco V. (2013). Pulmonary Function and Airway Responsiveness in Young Competitive Swimmers. Pediatr. Pulmonol..

[42] Meleski B.W., Malina R.M. (1985). Changes in Body Composition and Physique of Elite University-level Female Swimmers during a Competitive Season. J. Sports Sci..

[43] Kuberski M., Musial A., Choroszucho M. (2025). Longitudinal Effects of Swimming Training on Anthropometric Characteristics in Pre-Adolescent Girls. Phys. Act. Rev..

[44] Roelofs E.J., Smith-Ryan A.E., Trexler E.T., Hirsch K.R. (2017). Seasonal Effects on Body Composition, Muscle Characteristics, and Performance of Collegiate Swimmers and Divers. J. Athl. Train..

[45] Bielec G., Gozdziejewska A., Makar P. (2021). Changes in Body Composition and Anthropomorphic Measurements in Children Participating in Swimming and Non-Swimming Activities. Children.

[46] Kriemler S., Puder J., Zahner L., Roth R., Braun-Fahrländer C., Bedogni G. (2009). Cross-Validation of Bioelectrical Impedance Analysis for the Assessment of Body Composition in a Representative Sample of 6- to 13-Year-Old Children. Eur. J. Clin. Nutr..

[47] Fantuzzi G., Righi E., Predieri G., Giacobazzi P., Mastroianni K., Aggazzotti G. (2010). Prevalence of Ocular, Respiratory and Cutaneous Symptoms in Indoor Swimming Pool Workers and Exposure to Disinfection By-Products (DBPs). Int. J. Env. Res. Public Health.

[48] Tzelepis G.E., Vega D.L., Cohen M.E., McCool F.D. (1994). Lung Volume Specificity of Inspiratory Muscle Training. J. Appl. Physiol..

[49] Courteix D., Obert P., Lecoq A.-M., Guenon P., Koch G. (1997). Effect of Intensive Swimming Training on Lung Volumes, Airway Resistances and on the Maximal Expiratory Flow-Volume Relationship in Prepubertal Girls. Eur. J. Appl. Physiol..

[50] Davies R.D., Parent E.C., Steinback C.D., Kennedy M.D. (2018). The Effect of Different Training Loads on the Lung Health of Competitive Youth Swimmers. Int. J. Exerc. Sci..

[51] Stavrou V., Toubekis A.G., Karetsi E. (2015). Changes in Respiratory Parameters and Fin-Swimming Performance Following a 16-Week Training Period with Intermittent Breath Holding. J. Hum. Kinet..

